# Towards a New Assessment Tool for Caregivers of Patients with Disorders of Consciousness: The Social and Family Evaluation Scale (SAFE)

**DOI:** 10.3390/brainsci12030323

**Published:** 2022-02-28

**Authors:** Davide Sattin, Francesca Giulia Magnani, Martina Cacciatore, Matilde Leonardi

**Affiliations:** UOC Neurologia, Salute Pubblica, Disabilità, Fondazione IRCCS Istituto Neurologico Carlo Besta, 20133 Milan, Italy; davide.sattin@istituto-besta.it (D.S.); martina.cacciatore@istituto-besta.it (M.C.); matilde.leonardi@istituto-besta.it (M.L.)

**Keywords:** consciousness, assessment, measurement, caregivers, diagnosis

## Abstract

Monitoring the level of responsiveness of patients with Disorders of Consciousness (DoCs) represents an issue in all the settings where there is not the daily presence of clinicians, such as long-term and domestic settings. The involvement of patients’ informal caregivers (i.e., patients’ family) in such a monitoring process is thus fundamental. However, to date, no standardized tailored-made instruments exist that informal caregivers can use without the presence of clinicians, despite evidence illustrating the good accuracy of caregivers when expressing their opinions about the level of responsiveness of DoC patients. The present work aims to set the foundational knowledge, to create a standardized instrument that is able to assess the level of responsiveness of patients with DoCs by their informal caregivers. After selecting and modifying the items to be included in the new scale from the gold standard to diagnose DoCs (i.e., the Coma Recovery Scale-revised), and following a consensus process, we created the Social and Family Evaluation (SAFE) scale for caregivers of patients with DoCs. Although the SAFE needs a validation process, with the present work we provided its preliminary description along with insights into its clinical utility.

## 1. Introduction

The correct diagnosis of Disorders of Consciousness (DoCs) challenged clinicians for a long time and still represents one of the most complex processes for neuroscientists and clinicians. To date, the gold standard to diagnose DoCs, by distinguishing between a Vegetative State (VS; also known as Unresponsive Wakefulness Syndrome) and a Minimally Conscious State (MCS), is the Coma Recovery Scale-revised (CRS-r; [1]) consisting of 23 items, grouped into 6 subscales assessing different domains (auditory, visual, motor, oromotor, communication, and arousal). The CRS-r is fundamental for accurately classifying the level of responsiveness associated with consciousness. Having a gold standard tool to evaluate the responsiveness to external stimuli is also very important for other clinical reasons, such as treatment options and monitoring (e.g., decisions on pharmacological treatment with zolpidem [2]), prognostic evaluation, or to monitor behavioural responses to pain stimuli [3].

Given the high risk of misdiagnosis in DoCs, it has been proven that several CRS-r administrations can reduce this risk [4], although it is not clear yet what the optimal number of assessments is to reach a correct diagnosis [5]. As a consequence, the gold standard to diagnose DoCs is time-consuming and requires the specific expertise of clinicians to be correctly administered. Furthermore, the lack of daily presence of expert clinicians reduced the capacity to monitor the consciousness recovery in patients with DoCs who are in long-term care centers and at home, which is different from what happens in rehabilitation centers.

Recent studies highlighted how the presence of caregivers during clinical assessment can improve the performance level of the patients due to (i) their daily presence during the care pathway, (ii) their knowledge of the patient’s history, and (iii) the use of emotionally relevant stimuli (e.g., their voice), thus helping to reach the correct diagnosis [6,7,8]. Already in 1997, the Aspen Workgroup recommended the implementation of the caregivers’ observations in the assessment procedures whenever possible [9]. Moreover, the study by Sattin et al. [10] demonstrated that in 44.5% of the patients enrolled in their study (*n* = 92), the CRS-r total score was higher when the scale was administered by clinicians and caregivers altogether, compared to the CRS-r scores obtained when the scale was administered by trained clinicians only. The same study also revealed a change in diagnosis for 16% of the patients when considering the CRS-r score obtained from the clinicians along with caregivers’ administration of the scale [10]. Similarly, a recent study by Formisano et al. [11] highlighted how the inclusion of the caregivers during the CRS-r administration led to better responsiveness of patients, with higher scores mainly in auditory and motor domains [11].

Despite this evidence, it is difficult for clinicians to administer a series of CRS-r with caregivers. Indeed, several constraints do not allow the daily presence of caregivers in the hospital units (e.g., limited time available, work problems, other family to care for, etc.); moreover, caregivers often go to the hospital after work and it is difficult for clinicians to administer a CRS-r in the late afternoon. Furthermore, there is not a tailor-made instrument that caregivers can use without clinicians yet, although it is well known that caregivers are the persons who spend a lot of time with their patients (e.g., a study reported over 60% of the caregivers spending more than 3 h per day with their relatives [12]) and often are the first, together with the nursing staff, to recognize the first signals of recovery of consciousness [10,13].

So far, few attempts have been made to standardize the caregivers’ opinions about the DoC patients’ level of consciousness. For instance, Hermann et al. [14] proposed a new tool based on the feelings the formal caregivers (i.e., nursing staff members) have regarding the patients’ level of consciousness. Specifically, the tool consisted of a visual analogue scale (i.e., 100 mm length-line) administered to formal caregivers who quantified the best consciousness level observed given the question “le patient est-il là?” (i.e., “Is there anybody home?”). When compared with patients’ clinical diagnosis (i.e., VS or MCS), the tool demonstrated good accuracy in detecting the patients’ level of consciousness. However, it is worth noting that the caregivers in the study by Hermann et al. [14] did not include the informal caregivers, such as the familiars of DoC patients, who often are the persons who spend most of the time with the patients and have a better knowledge of the patients themselves. For this reason, also considering their perception about the patients’ consciousness level could be valuable. Indeed, in the study by Jox et al. [15], informal caregivers (family members) were asked to express their perception about the level of consciousness, which was compared with the clinical diagnosis. The authors found concordance between the caregivers’ perception and the clinical diagnoses (in 76% of cases, the relatives assumed the same level of consciousness indicated by diagnostic tests), stressing the importance to include the informal caregivers in the level of consciousness assessment processes. Similarly, Moretta et al. [16] asked forty-five informal caregivers to express their opinion about the level of patients’ environmental interaction as well as the patients’ ability to communicate through two questions requiring a yes/no response. The results highlighted that the totality of the caregivers of patients with MCS correctly perceived their ability to interact with the environment, whilst 45.4% of them (*n* = 10) wrongly attributed communication abilities to their patients. Similarly, caregivers of patients with a VS overestimate both the ability to interact with the environment (65.2%; *n* = 15) and communication abilities (21.7%; *n* = 5), probably attesting, in this case, how caregivers tend to judge the patients’ responsiveness more positively than professionals.

In any case, all of the above-mentioned studies asked a general opinion about the DoC patients’ abilities and consciousness level from the informal caregivers without exploring their perception in a structured way, considering discrete functional domains. Indeed, exploring the informal caregivers’ perception about different and well-circumscribed abilities of their patients could have a greater value in light of the need to monitor the patients’ level of functioning during the care pathway. Furthermore, to be validated, a tailor-made instrument for caregivers of patients with DoCs should be differently structured, matching already existing clinical instruments.

For these reasons, we propose to create a new tool based on the CRS-r scale (The Social and Family Evaluation scale, hereinafter called SAFE) that can be used by informal caregivers without clinicians to collect their opinion on patients’ functioning. This study aimed to reword the items linked to cognitive mediated behaviours to be included in the new assessment tool, taking the CRS-r as a reference scale, making it easier and more intuitive for DoC patients’ caregivers. In other words, we aimed at setting the foundational knowledge for the creation of a new assessment tool that can be used by the caregivers of DoC patients autonomously, after being accurately instructed by the clinicians. The tool will be particularly useful in those situations where it is impossible (or very difficult) to have a direct assessment of DoC patients by expert professionals.

## 2. Materials and Methods

The methodology for this work consisted of a three-step procedure including, (i) the items’ selection to be included in the new scale and their adaptation by a group of three neuropsychologists with experience with DoCs and DoC patients’ caregivers, (ii) a consensus process by a panel of multidisciplinary experts, and (iii) a final rating on the appropriateness of the items included in the final form of the scale by a group of three expert clinicians. The process adopted for points (ii) and (iii) did not conform to a Delphi method [17], as we asked two different panels to express their opinions concerning the re-wording (linguistically speaking) of the selected items, which are the same items of the CRS-r’s reference scale. After the responses of the first panel of experts, we asked the second panel of experts to express their agreement on the re-worded items, according to the responses of the first panel; hence, we looked for agreement on understandability.

### 2.1. SAFE Items’ Selection and Adaptation

The Social and Family Evaluation scale (SAFE) aims to collect caregivers’ opinions about the functional state of patients, considering different cognitive and sensory domains. To this aim, the items of the SAFE have been chosen by referring to the CRS-r items addressing different functional domains (i.e., visual, motor, auditory, oro-motor/verbal, and linguistic); we consider the CRS-r items indicating a diagnosis of MCS and emerging MCS (eMCS) only; in other words, we did not consider the items referring to reflexive behaviours indicative of VS diagnosis.

This process allowed us to create a draft version of the SAFE scale.

### 2.2. Consensus Process

#### 2.2.1. Participants

We recruited 7 participants characterized by at least 5 years of experience with DoC, though not in a clinical sense. In other words, we recruited a group of expert researchers in different disciplines (e.g., bioengineering, neurophysiology, and neuroradiology) who usually do not evaluate DoC patients with clinical scales at the bedside but who have knowledge on DoC functioning.

#### 2.2.2. Survey

Once we created a draft version of the SAFE, as described above, we set up a survey inquiring the group of experts on whether the items included in each subscale were clear enough (considering the simplicity and language familiarity of the target respondents, i.e., caregivers of DoC patients). Specifically, the survey was composed of 12 different questions consisting of rating the level of clarity of each item of the SAFE, along with instructions created for the caregivers on a 3-point Likert scale from 0 (i.e., not clear at all, to be modified) to 2 (i.e., clear, not to modify).

If the rating was equal to 0 and 1 for a specific item of the SAFE, we asked the experts to indicate the way to modify the item, including re-wording it.

### 2.3. Final Rating

After modifying the SAFE items according to the results obtained from the survey administered to the first group of experts, we asked 3 expert clinicians (i.e., with at least 10 years of experience, and familiar with CRS-r administration) to perform a final check on the appropriateness of the items included in the SAFE.

### 2.4. Statistical Analyses

Medians and Interquartile Ranges (IQR) were calculated for the first group of experts. We also computed the frequencies, expressed as percentages, considering the raters’ responses to each question. A cut-off of 50% was set to identify the items to be changed and re-worded.

## 3. Results

### 3.1. SAFE Items’ Selection

From the auditory subscale of the CRS-r, we selected only the “reproducible movements to command” and “consistent movement to command”, requiring both object-related and non-object-related movements.

From the visual subscale of the CRS-s, we selected 3 items corresponding to “visual fixation”, “visual pursuit”, and “object localization-reaching”. We did not select the “object recognition” item of the CRS-r visual subscale, as it elicits the same behaviour as “consistent” and “reproducible movement to command” object-related items of the auditory CRS-r subscale.

From the motor subscale, we selected 4 items corresponding to “localization to noxious stimulation”, “object manipulation”, “automatic motor response”, and “functional object use”.

From the oromotor-verbal subscale we selected only the “intelligible verbalization” item. While, from the communication subscale we selected 2 items, consisting of “non-functional intentional” and “functional appropriate”.

For each item, the patient’s caregiver is required to express whether, in the past 15 days, the patient manifested each specific behaviour answering “YES” or “NO” according to specific criteria, derived from the CRS-r.

### 3.2. Consensus Process

The median and IQR for each question of the survey are depicted in Table 1.

Considering the responses of the first group of experts, 5 items of the SAFE and the instructions for the caregivers have been re-worded, since more than 50% of the experts indicated them as not clear at all (i.e., score = 0) or not clear enough (i.e., score = 1), falling above the cut-off (Table 1).

### 3.3. Final Rating

The 3 expert clinicians agreed upon the final version of the items to be included in the SAFE, as they did not suggest any further changes.

## 4. Discussion

Despite the Coma Recovery Scale-revised (CRS-r [1]) represents the gold standard to diagnose Disorders of Consciousness (DoCs), multiple testing is needed to reduce the risk of misdiagnosis, thus making the CRS-r time-consuming. Furthermore, it is not always easily applicable, especially in long-term care centers and domestic settings, as it requires specific expertise to be administered. These features pose an issue in monitoring the level of consciousness of DoC patients over time in all those situations lacking the daily presence of expert clinicians (e.g., long-term care). It has been proven that the presence of patients’ family members (i.e., informal caregivers) during clinical evaluations can give rise to patients’ better responsiveness [4,10,11]. Both the UK [18] and European Academy of Neurology [19] guidelines on DoCs highlight the importance of including informal caregivers in the assessment process, as they have a better knowledge of patients’ history and their presence may increase chances of detecting responses [7,10,11,19]. However, the presence of patients’ informal caregivers during clinical evaluation is made difficult most of the time; furthermore, there is no existing instrument that the informal caregivers can use without the presence of the clinicians to assess the patients’ level of consciousness in a standardized way.

For these reasons, the aim of this study was to start the process leading to a new tool (i.e., the Social And Family Evaluation (SAFE) scale) for assessing the level of consciousness in patients with DoCs by their informal caregivers. The purpose of this process was to verify that the items were linguistically reformulated in the most comprehensible way (for informal caregivers), collecting the opinions of experts on the non-modification of the original nature (as derived from the CRS-r) of each item included in the SAFE.

In selecting the items of the SAFE, we considered the CRS-r as a reference scale. This choice is derived from the need to have a structured instrument that can be directly compared with the clinical scale usually adopted to diagnose DoCs. Therefore, we have only re-worded (i.e., in a linguistic sense) the items related to cognitively mediated behaviours, making them usable and comprehensible to caregivers.

Indeed, the few existing studies aimed at collecting the caregivers’ opinions about the functional state of DoC patients relied on very general questions on the level of consciousness [10,13,14,15,16] and communication abilities [16]. Although previous results demonstrated that caregivers’ opinions might ameliorate diagnostic accuracy, it is not yet clear whether inconsistencies between caregivers’ opinions on patients’ level of consciousness and clinical profile assessed through standard scales are the result of caregivers’ overestimating tendencies [16]. In 1997, a qualitative study explored the relationship between informal caregivers’ beliefs and the patients’ objective functional status, highlighting a tendency in positively estimating observed behaviours independently from their clinical meaning [20]. Possibly, in all these previous cases, there lacked a matching between what the clinicians considered to assess the level of consciousness (e.g., different behavioural levels in specific sensory domains) and the signs the informal caregivers relied on to express their opinions. We think that creating a matched instrument with the clinical scale usually adopted by clinicians could improve the usefulness of caregivers’ opinions, especially in long-term settings, for monitoring reasons. In other words, creating a structured instrument directing the caregivers on what they have to focus on in expressing their opinions increases the probability to compare their opinions to what emerges during clinical evaluation. This could be potentially useful to perform periodical checkpoints when standard clinical assessments cannot be performed (e.g., when the patient is in a domestic setting). Moreover, helping caregivers to focus their attention on the behaviours important for clinicians could be useful to create a common language between caregivers and professionals, increasing their alliance for the cure and care of patients.

We choose to consider the caregivers’ opinion based on the behaviours manifested by the patients in a given time-window (i.e., the past 15 days) rather than following a standard observational approach consisting of rating the behaviour after direct external stimulation. This has the advantage of not instructing the caregivers about the observational criteria and stimuli administration as well as on the criteria to quantifying what has been observed following external stimulation.

A limitation of our work lies in not having considered some items that are a matter of debate at the moment. Specifically, items including auditory localization [21], contingent behavior (e.g., affective response; see the CRS-R manual, updated in 2020), and resistance to eye opening [22]) can be viewed as signs of consciousness, and are thus not linked to VS/UWS diagnosis. The exclusion of these items from the SAFE was done because, at the beginning of our work, we decided to follow the original standard version of the CRS-r. However, depending on the international debate about the significance of these behaviours, the inclusion of further items in future versions of the SAFE will be considered.

We acknowledge that an observational approach following incidental external stimulation would have led to a better comparison with what is detected by the scales used in the clinical routine; however, we think that our approach is easier and less expensive in terms of both times and resources for both clinicians and caregivers. Furthermore, the standard observational approach would expose, in contrast, the risk of the underestimation of certain behaviours caught by the caregivers in habitual conditions. In any case, in the SAFE we implemented criteria directing caregivers towards what to consider in expressing their opinions, maintaining the reproducibility principle as in the CRS-r.

## 5. Conclusions

With the present study, we provided preliminary insights towards the creation of a specific tool helping clinicians to monitor the level of consciousness of patients with DoCs in all those situations that do not allow standardized clinical assessment over time, such as long-term care and domestic settings. The involvement of patients’ caregivers in this process is made fundamental as they have a better knowledge of patients’ history and sometimes are the only ones spending most of their time with patients. This article presents the preliminary results of a process, and the SAFE scale must be validated in the near future so as to make it available as soon as possible.

## Figures and Tables

**Table 1:** Median and Interquartile Range (IQR) are presented for each question of the survey. The percentage of respondents indicating each item as “Clear” (i.e., score = 2) or “To be changed” (i.e., cumulative percentage of scores 1 and 0) is also reported. Each question of the survey referred to specific items of the SAFE (see the main text for further details): Q1, instructions; Q2, visual subscale—visual fixation item; Q3, visual subscale—visual pursuit item; Q4, motor subscale—object manipulation item; Q5, motor subscale—automatic motor response item; Q6, oromotor-verbal subscale—intelligible verbalization item; Q7, communication subscale—non-functional intentional and functional appropriate items; Q8, motor subscale—noxious stimulation item; Q9, auditory subscale—non-object related reproducible movements to command and consistent movement to command items; Q10, auditory subscale—object-related reproducible movements to command and consistent movement to command items; Q11, visual subscale—object localization-reaching item; Q12, motor subscale—functional object use item.

	Q1	Q2	Q3	Q4	Q5	Q6	Q7	Q8	Q9	Q10	Q11	Q12
Median	1	2	1	1	1	2	1	1	2	2	2	2
IQR	0.75	1	1	1	1	1	0.5	1	1	1	1	0.5
Clear (%)	42.9	57.1	42.9	42.9	42.9	57.1	28.6	42.9	57.1	57.1	57.1	71.4
To be changed (%)	57.1	42.9	57.1	57.1	57.1	42.9	71.4	57.1	42.9	42.9	42.9	28.57

## Data Availability

Data available in an accessible repository on request.
